# Supplementary material for: Programmable photoacoustic patterning of microparticles in air
Source: Nat Commun. 2024 Apr 16;15:3250. doi: 10.1038/s41467-024-47631-8 (PMC11021490; doi:10.1038/s41467-024-47631-8)
Supplement: Supplementary file 1 — Supplementary information [file 41467_2024_47631_MOESM1_ESM.pdf]

Supplementary information of "Programmable Photoacoustic Patterning of Microparticles in Air"

Note 1. Theoretical derivation process of photoacoustic excitation of Lamb wave

The photoacoustic effect is a complex process involving the conversion of light energy into heat energy and subsequently into mechanical energy. The time scales of opto-thermal conversion and thermal-vibrational conversion differ significantly. Therefore, it is possible to separate these two energy conversion processes and consider them individually. In the derivation of the opto-thermal process, we initially calculated the temporal variation of the surface temperature of the multilayer membrane. Using the surface temperature as a boundary condition, we further calculated the heat diffusion within the membrane, resulting in the spatiotemporal distribution of temperature. By considering the strain caused by temperature changes, we determined the initial displacement. Treating the multilayer film under laser stripe illumination as an infinitely long beam, we applied the initial displacement to the vibration equation of the beam. This approach allowed us to ascertain the spatiotemporal diagrams that illustrate the deformation of the membrane. Throughout the derivation, we observed a linear relationship between the laser power density and both the temperature and vibration displacement of the multilayer membrane. The detailed derivation process of the equations is presented in S. Fig. 1.

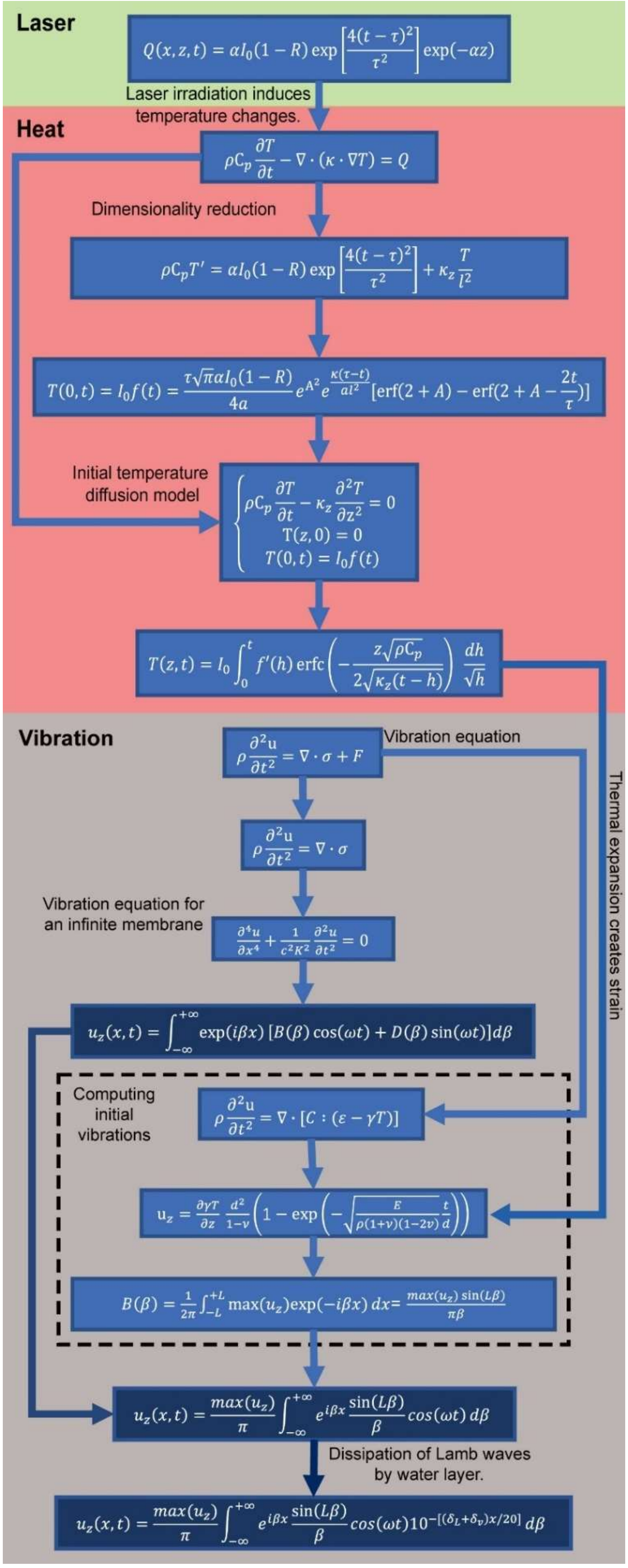

S. Fig. 1| Flowchart of the derivation process.

The numerical values of the physical quantities used in the derivation are summarized in S. Table 1

name	symbol	value	name	symbol	value
Reflectivity	R	0.41	Poisson ratio	ν	0.3
Thermal capacity	C_p	$500\, J/Kg\cdot m$	Young's modulus	E	$210\, GPa$
Pulse width	τ	$6\, ns$	Membrane thickness	d	$5\mu m$
Optical absorption coefficient	α	$7\times 10^7\, m^{-1}$	Sound velocity in water	c_f	$1490\, m/s$
Illumination width	$2L$	$400\, \mu m$	Water density	ρ_f	$1000\, Kg/m^3$
Thermal conductivity	κ	$45\, W/m\cdot K$	Temperature dissipation distance	l	$90\, nm$
Stainless-steel density	ρ	$7930\, Kg/m^3$	viscosity ratio of water	η	$1\times 10^{-3}\, Pa\cdot s$

S. Table 1|. Numerical values of physical quantities

We assume that the basic illumination unit is a $2L = 400\, \mu m$ wide line has a constant laser power in the Y direction. Therefore, we can express the laser power as follows:

$$I(z,t) = \begin{cases} I_0(1-R)e^{-\frac{4(t-\tau)^2}{\tau^2}}e^{-az} & (|x| < L) \\ 0 & (|x| \geq L) \end{cases} \quad (1)$$

where I is the laser power density, I_0 is the initial laser power density, R is the reflectivity of materials, τ is the pulse width, α is the optical absorption coefficient, z is the penetration depth of laser. We simplified the multilayer membrane as a 5 μm stainless-steel material with a light energy absorption ratio equivalent to that of TiN.

We can calculate the temperature spatial-time distribution in multilayer membrane through the thermal conduction equation expressed as follows, where Q represents the input heat, which in this section is supplied by the absorbing laser energy [1].

$$\rho C_p \frac{\partial T}{\partial t} - \nabla \cdot (\kappa \cdot \nabla T) = Q \tag{2}$$

where ρ is the density, C_p is the thermal capacity and κ is the thermal conductivity of stainless-steel. The laser light source is uniform in the x-direction within the region of $|x| < L$, meaning that the resulting temperature is also uniformly distributed in the x-direction within that region. Therefore, we can rewrite the above equation into the following one-dimensional form:

$$\rho C_p \frac{\partial T}{\partial t} - \kappa \frac{\partial^2 T}{\partial z^2} = \begin{cases} aI_0(1-R)e^{-\frac{4(t-\tau)^2}{\tau^2}} e^{-az} & (|x| < L) \\ 0 & (|x| \geq L) \end{cases} \tag{3}$$

Given the high optical absorption coefficient of stainless steel, the laser penetration depth in the membrane is shallow. As a result, we can assume that only the surface absorbs the laser energy, leading to a localized increase in temperature that subsequently diffuses into the interior of the multilayer membrane. Equation (3) can be reformulated as follows:

$$\rho C_p \frac{dT}{dt} = \alpha I_0 \, (1-R)e^{\frac{4(t-\tau)^2}{\tau^2}} + \kappa \, \frac{T}{l^2} \quad (|x| < L) \tag{4}$$

where l is the temperature dissipation distance. By solving the above differential equation, we can obtain the variation of the surface temperature of stainless steel with respect to time:

$$T(0,t) = \frac{\tau\sqrt{\pi}\alpha I_0\,(1-R)}{4a} e^{A^2}\ e^{\frac{\kappa(\tau-t)}{al^2}}\ [erf(2+A) - erf(2+A-\frac{2t}{\tau})] \tag{5}$$

where $a = \rho C_p$, $A = \frac{\tau\kappa}{4al^2}$ and $erf(X)$ is error function. At this point, the problem transforms into a classical heat diffusion problem with temperature boundary conditions:

$$\begin{cases} \rho C_p \dfrac{\partial T}{\partial t} - \kappa_z \dfrac{\partial^2 T}{\partial z^2} = 0 \\ T(z,0) = 0 \\ T(0,t) = I_0 f(t) \end{cases} \tag{6}$$

where $f(t) = \frac{\tau\sqrt{\pi}\alpha\,(1-R)}{4a} e^{A^2}\ e^{\frac{\kappa(\tau-t)}{al^2}}\ [erf(2+A) - erf(2+A-\frac{2t}{\tau})]$.

The solution takes the following form:

$$T(z,t) = I_0 \int_0^t f'(h) \cdot erfc\left(-\frac{z\sqrt{\rho C_p}}{2\sqrt{\kappa_z(t-h)}}\right)\frac{dh}{\sqrt{h}} \tag{7}$$

where $erfc(X)$ is co-error function. We compared the spatiotemporal distribution of the temperature field obtained from the solution with the simulated results by COMSOL, and the comparison is shown in S. Fig. 2a and 2b. In this comparison, the laser power density used is $1\ MW/cm^2$.

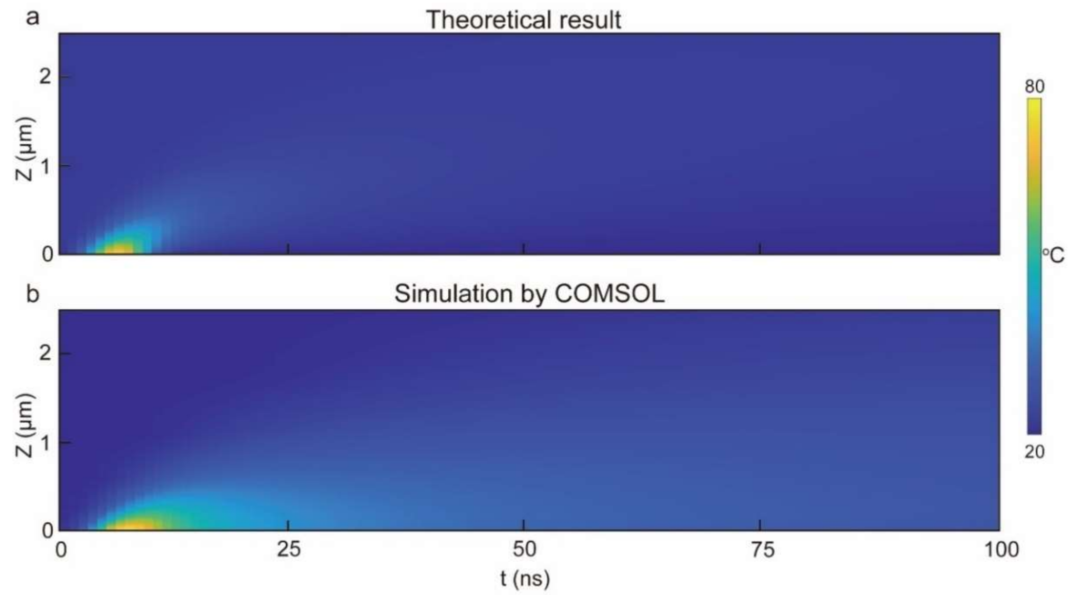

S. Fig. 2|Space-time distribution of the temperature field obtained from the theoretical solution and the simulated results. a, Theoretical results depicting space-time diagrams of temperature induced by the photothermal effect. **b**, Simulation results depicting space-time diagrams of temperature induced by the photothermal effect.

Based on the aforementioned figure, it is evident that the temperature variation primarily occurs on the surface of the multilayer membrane and dissipates rapidly. This observation provides valuable insights for deriving the vibration field of the metallic membrane. The equation of motion in a solid medium can be expressed as follows [2]:

$$\rho \frac{\partial^2 u}{\partial t^2} = \nabla \cdot \sigma + F \tag{8}$$

where u is the displacement, σ is stress and F is external force which oriented from thermal expansion in this section. As the rapid temperature change occurs within a brief duration of 10 ns, which is significantly shorter compared to the vibration dynamics, we can treat the effects of thermal expansion as an initial condition rather than incorporating external forces into the differential equation. Considering the specific nature of the system in this paper, we can make certain approximations. Since the thickness of the multilayer membrane is much smaller than the possible wavelength of

vibrations, and its width is much larger than the possible wavelength of vibrations, the entire system can be approximated as the vibration of an infinitely long slender beam under impact. The vibration equation governing the transient behavior of an infinitely long slender beam can be expressed as follows:

$$\frac{\partial^4 u_z}{\partial x^4}+\frac{1}{c^2K^2}\frac{\partial^2 u_z}{\partial t^2}=0 \tag{9}$$

where u_z is the displacement in Z direction, $c=\sqrt{\frac{E}{\rho}}$ is the longitudinal wave velocity, E is Yang's modulus and $K=\frac{d}{\sqrt{12}}$ is the sectional radius of gyration of a rectangular shape with a thickness of d. The solutions of equation (9) is

$$u_z(x,t)=\int_{-\infty}^{+\infty} e^{i\beta x}[B(\beta)\,cos(\omega t)+D(\beta)\,sin(\omega t)]d\beta \tag{10}$$

where ω is angular frequency, $\beta=\sqrt{\frac{\omega}{cK}}$ is spatial frequency, $B(\beta)=\frac{1}{2\pi}\int_{-\infty}^{+\infty} e^{-i\beta x}u(x,0)dx$ and $D(\beta)=\frac{1}{2\pi\omega}\int_{-\infty}^{+\infty} e^{-i\beta}\ v(x,0)dx$. $u(x,0)$ and $v(x,0)$ are initial displacement and velocity respectively. At this stage, it is necessary to determine the initial motion state of the multilayer membrane. Considering that the expansion of an object due to heating corresponds to a change in strain, equation (8) should be reformulated as follows [3]:

$$\rho\frac{\partial^2 u}{\partial t^2}=\nabla\cdot[C:(\varepsilon-\gamma T)] \tag{11}$$

where C is elasticity tensor, ε is strain tensors, γ is the thermal expansion tensor. Let's rearrange equation (11) in terms of displacement as the variable:

$$\begin{cases} \rho \dfrac{\partial^2 u_x}{\partial t^2} = \dfrac{E(1-\nu)}{(1+\nu)(1-2\nu)} \dfrac{\partial^2 u_x}{\partial x^2} + \dfrac{E\left(\frac{\nu}{1-2\nu}+\frac{1}{4}\right)}{(1+\nu)} \dfrac{\partial^2 u_z}{\partial x \partial z} + \cdots \\ \dfrac{E}{4(1-\nu)} \dfrac{\partial^2 u_x}{\partial z^2} - \dfrac{E}{1+\nu(1-2\nu)} \dfrac{\partial \gamma T}{\partial x} \\ \rho \dfrac{\partial^2 u_z}{\partial t^2} = \dfrac{E(1-\nu)}{(1+\nu)(1-2\nu)} \dfrac{\partial^2 u_z}{\partial z^2} + \dfrac{E\left(\frac{\nu}{1-2\nu}+\frac{1}{4}\right)}{(1+\nu)} \dfrac{\partial^2 u_x}{\partial x \partial z} + \cdots \\ \dfrac{E}{4(1-\nu)} \dfrac{\partial^2 u_z}{\partial x^2} - \dfrac{E}{(1+\nu)(1-2\nu)} \dfrac{\partial \gamma T}{\partial z} \end{cases} \tag{12}$$

where ν is Poisson ratio. Since u_x is much smaller than u_z, we neglect the effect of u_x here. The equation is rearranged as follows:

$$\rho \frac{\partial^2 u_z}{\partial t^2} = \frac{E(1-\nu)}{(1+\nu)(1-2\nu)} \frac{\partial^2 u_z}{\partial z^2} - \frac{E}{(1+\nu)(1-2\nu)} \frac{\partial \gamma T}{\partial z} \tag{13}$$

Solving the above equation yields:

$$u_z = \frac{\partial \gamma T}{\partial z} \frac{d^2}{1-\nu} \left(1 - exp\left(- \sqrt{\frac{E}{\rho(1+\nu)(1-2\nu)}} \frac{t}{d} \right) \right) \tag{14}$$

The spatial-temporal distribution of displacement obtained does not account for the propagation of vibrations in the x-direction, thus it remains relatively accurate only for the initial tens of nanoseconds. To address this, we can utilize the maximum displacement from the distribution as the initial displacement function and substitute it into equation (10):

$$u_z(x,t) = \frac{max(u_z)}{\pi} \int_{-\infty}^{+\infty} e^{i\beta x} \frac{\sin(L\beta)}{\beta} cos(\omega t)\, d\beta \tag{15}$$

In the experiment, there exists a water layer beneath the multilayer membrane,

with an approximate thickness of 2 cm. The presence of this water layer introduces impedance to the vibration, and the level of impedance is dependent on the frequency of the vibration. There are two main forms of attenuation for Lamb waves in water: viscous attenuation δ_L and leaky wave attenuation δ_v, The attenuation coefficient is given by [4]:

$$\delta_L = \frac{2\omega^2\eta}{3c_f^3\rho_f} \tag{16}$$

$$\delta_v = \frac{1}{2d}\sqrt{\frac{\omega\eta\rho_f}{2E\rho_s}} \tag{17}$$

where η is viscosity ratio and c_f is velocity and ρ_f is density of fluid. Taking into account the attenuation of lamb wave, the displacement equation is given by:

$$u_z(x,t) = \frac{\max(u_z)}{\pi}\int_{-\infty}^{+\infty} e^{i\beta x}\frac{sin(L\beta)}{\beta}cos(\omega t)\cdot 10^{-\left[\frac{(\delta_L+\delta_v)x}{20}\right]}d\beta \tag{18}$$

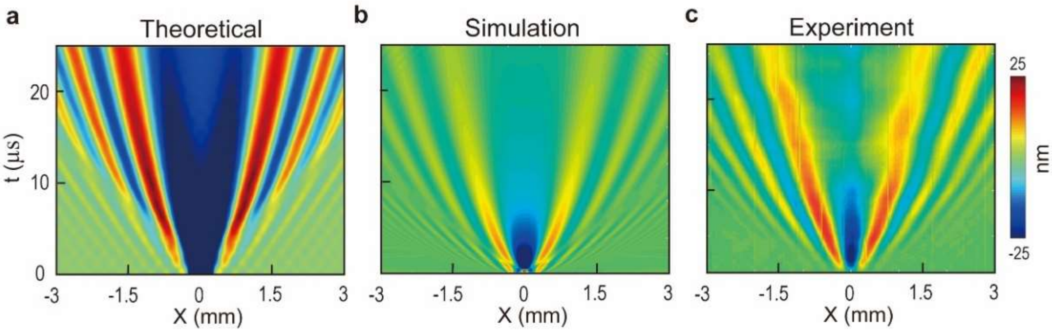

S. Fig. 3| a, Theoretical calculation of elastic wave propagation depicted in space-time diagrams. **b**, Numerical simulation of elastic wave propagation depicted in space-time diagrams. **c**, Experimental measurements of elastic wave propagation depicted in space-time diagrams.

S. Fig. 3a. b and c illustrate the theoretical result, simulation and experimental measurements of the membrane's deformation in z direction upon the illumination of the pulsed laser at a power density of 1.0 MW/cm^2. demonstrate the overall consistency of the results obtained from the three methods. This verification confirms the validity of our theoretical deductions. As illustrated in S. Fig. 3, the pulsed laser excites localized Lamb waves within the thin film, which gradually propagate outwards over time. These localized Lamb waves have the capability to displace particles from the thin film, allowing them to arrange themselves into arbitrary patterns.

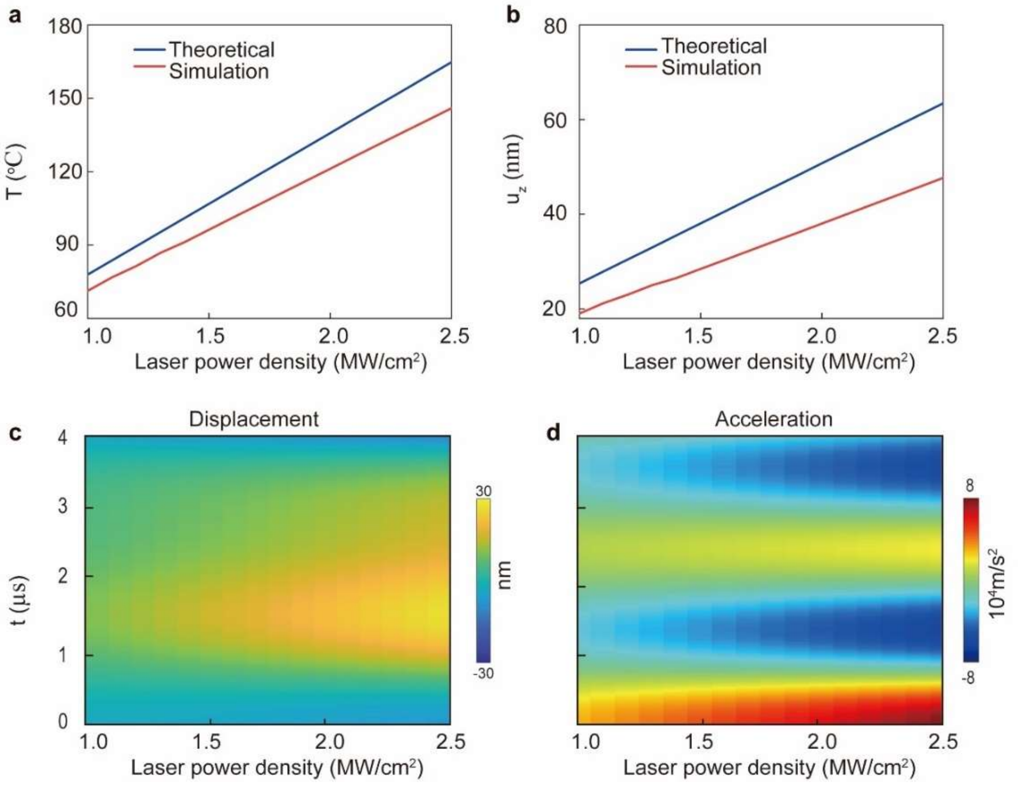

S. Fig. 4| a, The maximum temperature of the multilayer membrane varies with changes in laser power density. **b**, The maximum displacement of the multilayer membrane varies with changes in laser power density **c**, Theoretical displacement of the multilayer membrane at x = 0.5 mm exhibits variations with changes in both time and laser power density. **d**, Theoretical acceleration of the multilayer membrane at x = 0.5 mm exhibits variations with changes in both time and laser power density.

During the derivation of the formulas, it is evident that the intensity of the laser has a linear impact on both the temperature increase of the metal membrane and the intensity of vibration in the metal membrane.

S. Fig. 4a illustrates the relationship between the light intensity and the calculated maximum temperature increase in the membrane, comparing the theoretical and simulated results. Similarly, S. Fig. 4b displays the relationship between the laser intensity and the calculated maximum vibration displacement in the membrane, again comparing the theoretical and simulated results. These findings align with our initial expectations. It is important to note that despite the temperature within the membrane exceeding 100 °C, it rapidly dissipates to room temperature and does not reach a boiling point that would affect the water layer.

In S. Fig. 4c, we presented the variations of displacement with respect to time and laser power density at the coordinate x = 0.5 mm. Additionally, in S. Fig. 4d, we displayed the corresponding variations of acceleration. It is observed that the vibration field at the point located at coordinate x = 0.5 mm on the membrane demonstrates a linear correlation with the incident laser power density at all time instances.

The silica particles positioned on the multilayer membrane undergo distinct force states as a result of the varying motion of the membrane. This phenomenon is depicted in S. Fig. 8, which presents a schematic diagram illustrating the concept.

Note 2. Simulation of photoacoustic excitation of Lamb wave

The theoretical derivation involved certain approximations, leading to some differences between the results and the experimental data. In comparison, the simulation results obtained using finite element differential methods are closer to the experimental observations. The parameters used in the simulation are consistent with those used in the theoretical derivation.

The temporal evolution of various physical processes under the Laser power density of 2.0 MW/cm^2 is shown in S. Fig. 5. We observed that following the 6ns laser pulse, the temperature of the multilayer membrane reaches its peak at 20 ns and quickly returns to room temperature in less than 1 μs. The temperature rise induces thermal expansion, leading to the propagation of Lamb waves in the multilayer membrane. The vibration at the x = 0 mm position ceases after 10 μs. Subsequently, the multilayer membrane impacts the particles, causing them to be dislodged. The flight time of the particles is within 10 ms. The pulse interval of 100 ms in our experiment is long enough for the system to return to the initial configuration. As a result, the dynamic assembly process of the particles after each pulse is independent and does not influence one another.

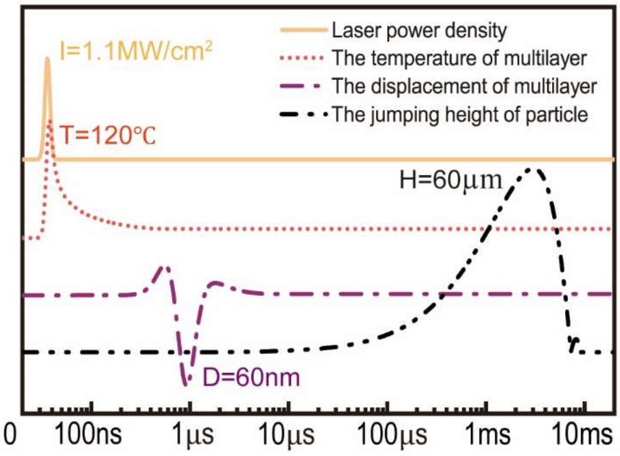

S. Fig. 5| Simulation of elastic waves induced by photoacoustic effect. Illustration of various parameters: the transient laser intensity of the multilayer surface, the corresponding temperature rise in the multilayer, the displacement of the multilayer under the influence, and the resulting particle motions.

Gaining vibration information from the multilayer membrane is advantageous for studying the corresponding arrangement behavior of the particles. Initially, the particles are attached to the multilayer membrane and share the same motion state. The detachment process occurs when the particles' acceleration becomes lower than the acceleration of the metal membrane, causing the distance between the particles and the

membrane to increase. The simulation displacement and velocity of the coordinate (x = 0.5 mm) with time shown in the S. Fig. 6a. The time range for which both displacement and velocity are positive is approximately between 0.6 μs and 2.2 μs. And the simulation acceleration of this point is shown in S. Fig. 6b and the green translucent rectangle encompasses the area where acceleration is negative. S. Fig. 6c depicts the displacement and velocity of the point (x = 0.5 mm) over time in experimental situation. The time range for which both displacement and velocity are positive is roughly between 0.5 μs and 2.2 μs. At this moment, if the particle's acceleration is lower than that of the multilayer, the particle will detach from the multilayer and undergo a process of jumping and falling.

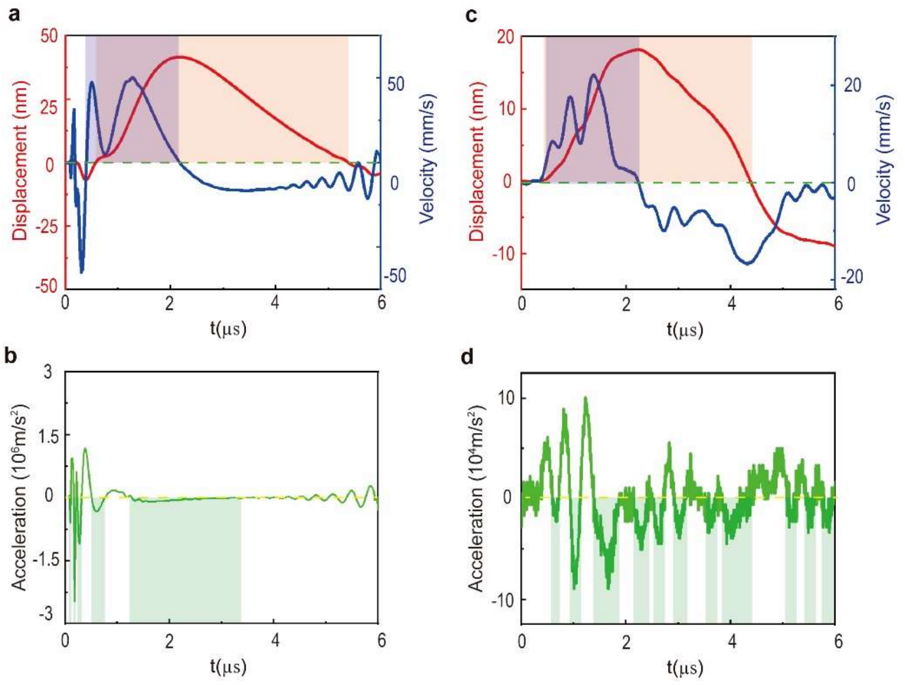

S. Fig. 6| Simulated and experimental time-dependent vibration at x = 0.5 mm a, Simulated displacement represented by red line and velocity represented by blue line. **b**, Simulated acceleration represented by green line. **c**, Experimental displacement and velocity with time. **d**, Experimental acceleration with time.

Note 3. Effects of adhesive force between particles and the substrate

On the surface of the plate, various forces act on the microparticles, with van der Waals and electrostatic forces playing a dominant role. In this work, we use particles with a diameter of 25 μm. The graphite layer coated on the membrane significantly weakens the electrostatic force acting on the particles. Hence, the dominant force between micrometer-sized particles and a flat surface is the van der Waals force, which is tens of thousands of times stronger than the force of gravity acting on the particles.

The formula for van der Waals forces between spherical particles and a flat surface is as follows [5, 6]:

$$F_{vdw} = \frac{A \cdot r}{6d^2} + \frac{A}{6\pi d^3} \cdot \pi z^2 \tag{19}$$

where the A represents the Hamaker constant, r represents the particles' radius, d represents the separation distance between the particle and the surface, and z is the radius of the contact area between the particles and the plate. In fact, it is the existence of pressure that allows the particles to reach a balance of forces. The formula for pressure relative to deformation is:

$$z = \sqrt[3]{3 \cdot F_{def} \cdot r/4E'} \tag{20}$$

where the F_{def} represents the deformation pressure, $E' = 1/\left(\left(\frac{1-v_1^2}{E_1}\right) + \left(\frac{1-v_2^2}{E_2}\right)\right)$ represents the equivalent elastic modulus. E_1 and E_2 represents the yang's modulus of particles and flat surface, v_1 and v_2 represents the passion ratio of particles and flat surface.

Using these two formulas, we can calculate the deformation and adhesion force of particles of different sizes when they are statically placed on the multilayer membrane, as shown in S. Tab. 2.

	25 μm silica particle	40 μm silica particle	55 μm silica particle
z	161.58 nm	244.80 nm	325.01 nm
G	−0.14 nN	−0.59 nN	−1.54 nN
F_{vdw}	−46.58 μN	−55.95 μN	−95.50 μN
F_{def}	46.58 μN	55.95 μN	95.50 μN

S. Tab. 2| The Comparison of gravity and Van der Waals force of silica particles with diameters of 25 μm, 40 μm, and 55 μm. By convention, we define the upward direction as the positive direction. To differentiate forces acting in the downward direction, we assign a negative sign to them.

In S. Fig. 7, we compared the quality of ring patterns formed by different particles under a laser power density of 1.5 MW/cm^2. For S. Fig. 7a, we found that particles with smaller sizes, while having the same shape, materials, power density, and exposure time, exhibited lower adhesion forces and higher patterning quality. For S. Fig. 7a3, b2, and c, despite the significant difference in densities among the three types of particles: silica particles (2000 kg/m^3), zirconia particles (6000 kg/m^3), and pollen (1000 kg/m^3), the patterning results are similar. In S. Fig. 7b3, the zirconia particles have a diameter of up to 100 μm and a relatively high density. The gravitational force acting on these particles can be comparable to the van der Waals force. Due to the lower static friction experienced by spherical particles, the particles can undergo more random rolling on the membrane. Indeed, when the particles are non-spherical, the van der Waals forces between the particles and the membrane can increase significantly due to the larger contact area. As seen in Fig. 7d, quartz sand, which has a similar material composition to silica particles but larger particle size, exhibits challenges in improving the quality of pattern formation even with four times the action time. We found that improving the pattern formation quality can be achieved by increasing the laser power density.

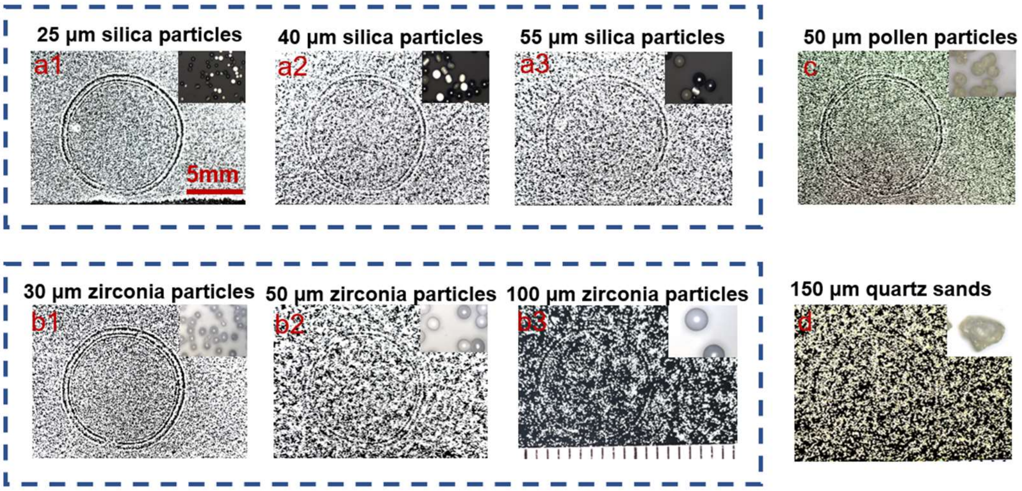

S. Fig. 7| The impact of particle types on pattern formation. a, Comparison of the quality of ring patterns formed by silica particles with diameters of 25 μm, 40 μm, and 55 μm under a laser power density of 1.5 MW/cm^2 in 3 s. **b**, Patterns formed by zirconia particles with diameters of 30 μm, 50 μm, and 100 μm. **c**, Patterns formed by 50 μm pollen particles. **d**, Patterns formed by 150 μm quartz sands under a laser power density of 1.5 MW/cm^2 in 12 s.

Note 4. Simulation of motion analysis of particles

During the stage represented by S. Fig. 8a-d where the particles vibrate in sync with the multilayer membrane, the net force acting on the particles can be determined by considering the acceleration of the multilayer membrane and the mass of the particles. In S. Fig. 9, the red line represents the net force acting on the particle located at the coordinate x = 0.5 mm. The corresponding laser power density in this case is 2.0 MW/cm^2. The detachment point occurs between S. Fig. 8d and S. Fig. 8e, when the upward velocity of the membrane reaches its maximum. Subsequently, both the membrane and particle velocities decrease. However, if the membrane decelerates faster than the particles, the distance between the membrane and particles will increase.

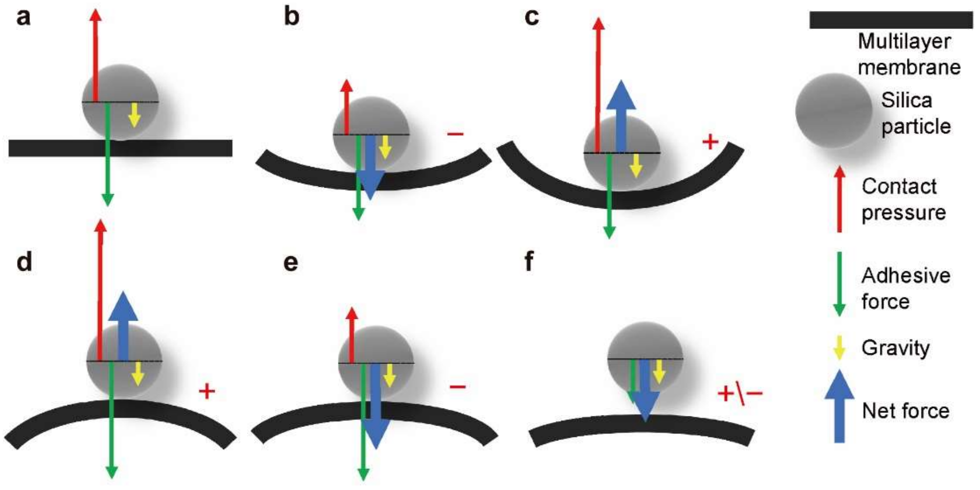

S. Fig. 8| The force state of a silica particle on the multilayer membrane. The positive and negative signs in red represent the direction of acceleration of the multilayer membrane, with upward indicating the positive direction. **a**, Net force acting on the particles is zero in stationary state. **b**, Net force acting on the particles is directed downward when multilayer membrane is accelerating downward. **c-d**, Net force acting on the particles is directed upward when acceleration of multilayer membrane is upward. **e**, Net force acting on the particles is directed downward, and the distance between the particles and the membrane increases during this stage. **f**, Net force is solely provided by gravity and contact pressure as the distance continues to increase.

As shown in S. Fig. 8e-f, when the particle begins to detach from the multilayer membrane, the pressure exerted on the particles by the multilayer membrane gradually decreases, and the acceleration of the particles remains negative throughout this process. To calculate the net force acting on the particles, the distance between the particles and the membrane needs to be known. We employed numerical integration to calculate the motion of the particles during the detachment process, and the net force on the particles can be determined. The calculated result after detachment is represented by the blue line in S. Fig. 9.

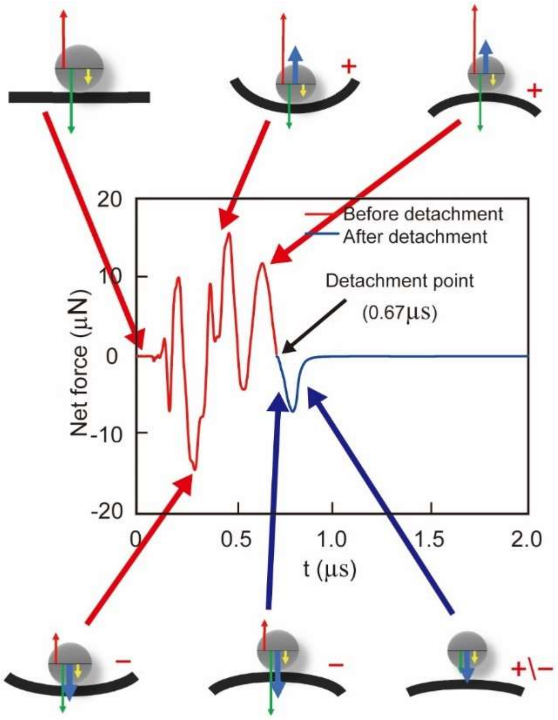

S. Fig. 9| Net force acting on particles at the coordinate x = 0.5 mm.

We utilize numerical integration methods to calculate the acceleration, velocity, and displacement of the particles. The process can be divided into two stages: the detachment stage and the ejection stage.

During the detachment stage, the main forces acting on the particles are the van der Waals force and the deformation pressure exerted by the multilayer membrane. Based on Note 1, for a particle located at $x = 0.5$ mm, the critical time for detachment is determined to be 0.67 μs. The time step of numerical integration is sited to 1 ns.

As the displacement difference between the particles and the multilayer membrane increases, the deformation pressure rapidly decreases, while the van der Waals force decreases more slowly but eventually approaches zero. This can be observed from S. Fig. 10a, where the downward acceleration of the particles initially increases, then decreases, and eventually approaches zero. At this point, we define the velocity of the particles as the detachment velocity. This means that at this velocity, the particles are no longer influenced by the multilayer membrane.

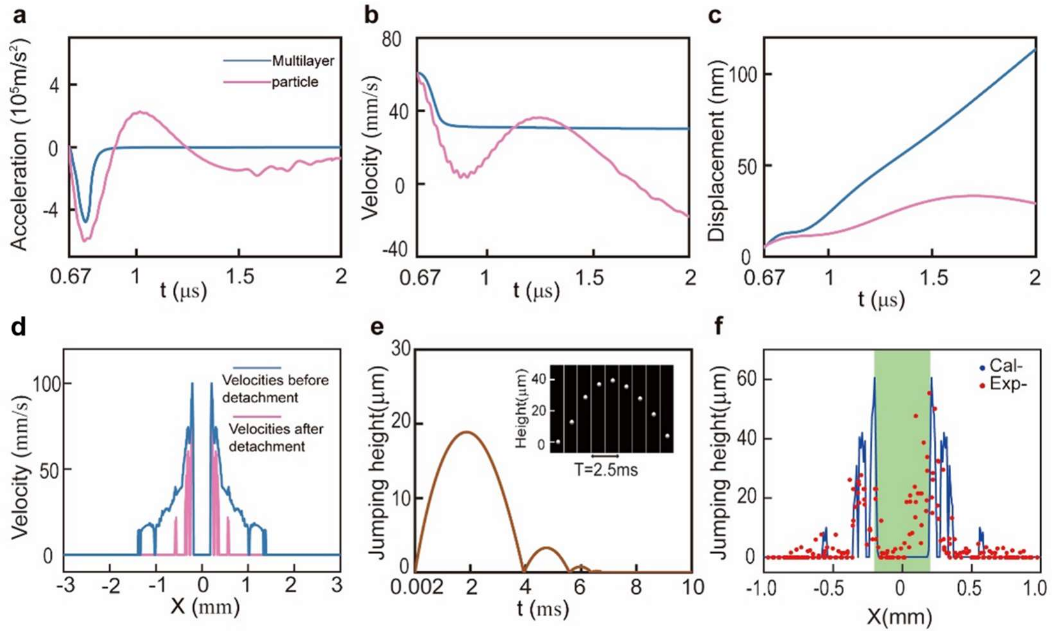

S. Fig. 10| Calculation of motion analysis of particles. a, Z-axis acceleration variation of the particle and the multilayer membrane at x = 0.5 mm in the detachment stage. **b**, Z-axis velocity variation of the particle and the multilayer membrane at x = 0.5 mm in the detachment stage. **c**, Z-axis displacement variation of the particle and the multilayer membrane at x = 0.5 mm in the detachment stage. **d**, Spatial distribution of particles velocities before and after the detachment stage. **e**, Z-axis displacement variation of a particle at x = 0.5 mm in the ejection state is shown. The inset images captured during the experiment demonstrates particles being ejected upwards at x = 0.25 mm. **f**, Relationship between maximum jumping heights and horizontal position of particles. calculation results represented by blue curve; experiment results represented by red dots. The light green rectangle area represents the laser illuminated region.

Subsequently, the ejection stage occurs within the following 10 ms. The time step of this stage is sited to 0.5 μs. The motion state of the particles at this stage is primarily influenced by gravity and air resistance. When considering air resistance under high Reynolds number conditions, the formula for air resistance can be expressed as:

$$f = \frac{1}{2} C_d \rho \cdot v^2 \cdot S_p \ , \qquad (6)$$

where the C_d represented the drag coefficient of particles, ρ represented the density of particles, v is the relative velocity between the particles and the air and $S_p = \pi \cdot r^2$ is the cross-sectional area of the particles.

The motion state of the particles at this stage is shown in S. Fig. 10e. By analyzing the motion of the particles, we can obtain the spatial distribution of the jumping heights across the multilayer membrane surface. This distribution is represented by the blue curve in S. Fig. 10f, and it demonstrates an agreement with the experimental results indicated by the red dots.

Note 5. The impact of parameter changes on the patterning effect

In S. Fig. 11a, the spatial distribution of the displacement in the Z-axis of the membrane is depicted, showcasing how it varies with changes in laser power density. It is evident from the figure that there is a positive correlation between the laser power density and the vibration displacement of the multilayer membrane. Additionally, we simulated the jumping height of particles under different laser power densities.

Like the calculation method in Note. 4, we obtained the spatial distribution of detachment velocities of particles, which is shown in S. Fig. 11b. Then, we can calculate the maximum jumping height of the particle varies with the changes of the laser power density. In S. Fig. 11c, the calculation results represented by green semicircles slightly differ from the experimental results represented by the blue dots, which may be due to the lack of consideration for camera's measurement error and particle rotation during the jumping process. It is evident that a higher power leads to better arrangement results.

This conclusion is also supported by patterning results, as depicted in S. Fig. 11d, which shows the arrangement of circular patterns with a size of 1 cm under different laser powers.

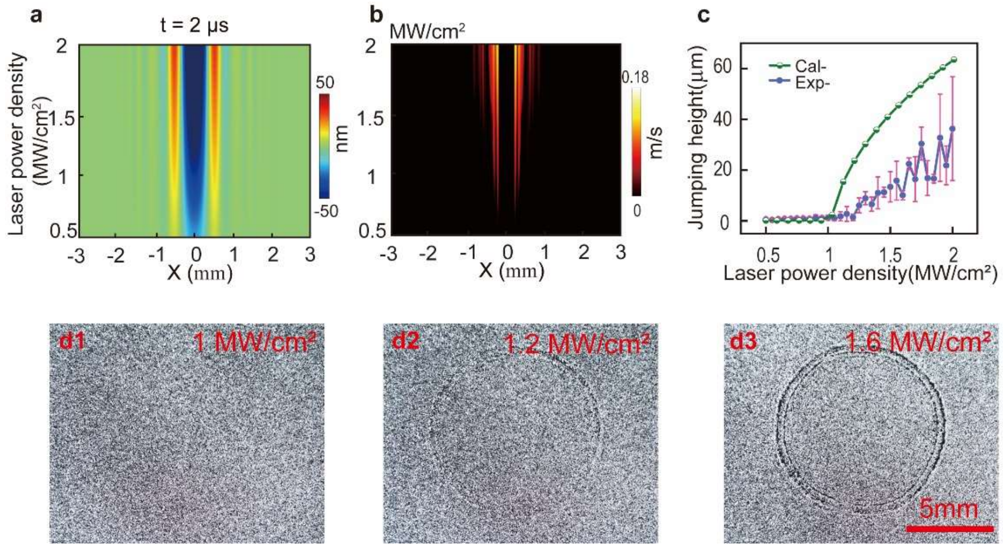

S. Fig. 11| Impact of laser power density on the quality of particle patterning. a, Spatial distribution of the vibration displacement of the multilayer membrane varies with changes in laser power density. **b,** Spatial distribution of particles detachment velocities varies with changes in laser power density. **c,** Relationship between particles' jumping height and laser power density. The green semicircle represents the simulation results, while the blue circle represents the experimental results. **d,** Comparison of three particle patterning results under three different laser power densities.

A simulation was performed to analyze the vibration of the multilayer membrane under different widths of laser stripes. As shown in S. Fig. 12a, a wider laser stripe results in stronger vibrations of the multilayer membrane and a wider area of vibration. Specifically, when the width of the laser stripe exceeds 400 μm, the vibrations from both sides of the line overlap, leading to significantly stronger vibrations in the central area around $x = 0$ mm.

Like the calculation method in Note. 3, we have obtained the spatial distribution of particles detachment velocities, as depicted in S. Fig. 12b. In S. Fig. 12c, we present the relationship between pattern resolution and the width of the laser stripe. In S. Fig. 12c, it is observed that increasing the width of the laser stripe results in a decrease in pattern resolution.

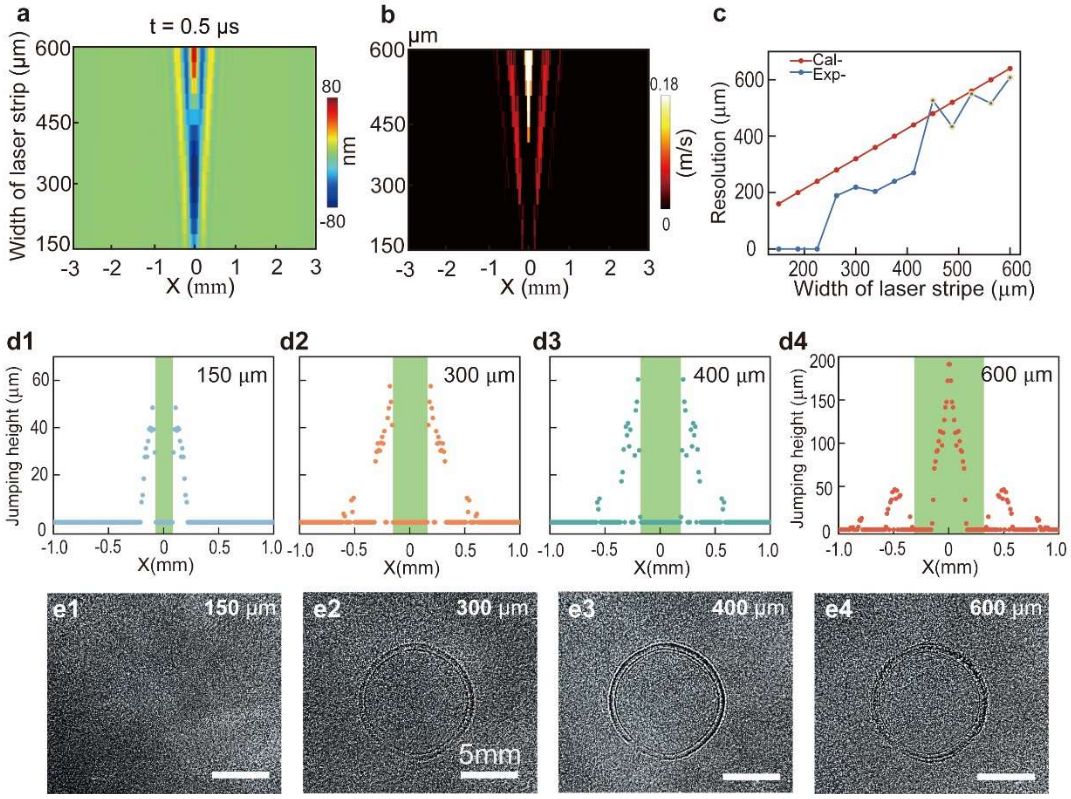

S. Fig. 12| Relationship between patterned resolution and laser stripe width. a, Spatial distribution of the vibration displacement of the multilayer membrane varies with changes in width of laser stripe. **b,** Spatial distribution of particles detachment velocities varies with changes in width of laser stripe. **c,** Relationship between patterned resolution and width of laser stripe. Blue dots represent calculation results, while red dots represent experiment results. **d,** maximum jumping heights of particles at corresponding positions are compared for laser stripe widths. The light green rectangle area represents the laser illuminated region. **e,** Comparison of patterned effects is shown with different laser stripe widths at a laser power density of 1.5 MW/cm².

It is apparent from S. Fig. 12d that the positions of the jumping particles on both sides are more localized but less clear when the width of the laser stripe are 150μm and 300 μm, in comparison to the situation that the width of laser stripe is 400 μm. Furthermore, when the width of the laser stripe is increased to 600 μm, the particles located in the center of the stripe exhibit the ability to jump up to a height of 200 μm.

This leads to the formation of a dark pattern in the central region, accompanied by an increase in the width of the pattern.

The calculations are verified by patterning results, as depicted in S. Fig. 12e. Laser stripe with width of 150 μm are less effective in exciting Lamb waves, while laser stripe with width of 600 μm cause multiple peaks and troughs on the membrane, resulting in the formation of thinner white lines.

Note 6. The theoretical explanation of dynamic flow

To provide a clear explanation of the dynamic flows, we conducted simulations considering gravitational force, air friction, and particle collision. As shown in S. Fig. 13, we simulate the behavior of 20 silica particles with diameter of 25 μm, spanning from 0.1 mm to 1.6 mm. The laser stripe, with a width of 400μm, moves from $x = 0.0$ mm to $x = 4.0$ mm over a 16 seconds duration in one cycle. The temporal-spatial positions of the 20 particles in the X direction were recorded every 4 seconds. The color shading at each data point indicates the number of particles at that position. It is can be observed that after 8 cycles (128 seconds), the particles tended to move towards positions near $x = 4.0$ mm.

This simulation provides an explanation for the dynamic flow of particles driven by a laser stripe. Additionally, in Fig. 4b, we demonstrate that periodic lines are more efficient than a single line, facilitating the sieving of particles to the desired location.

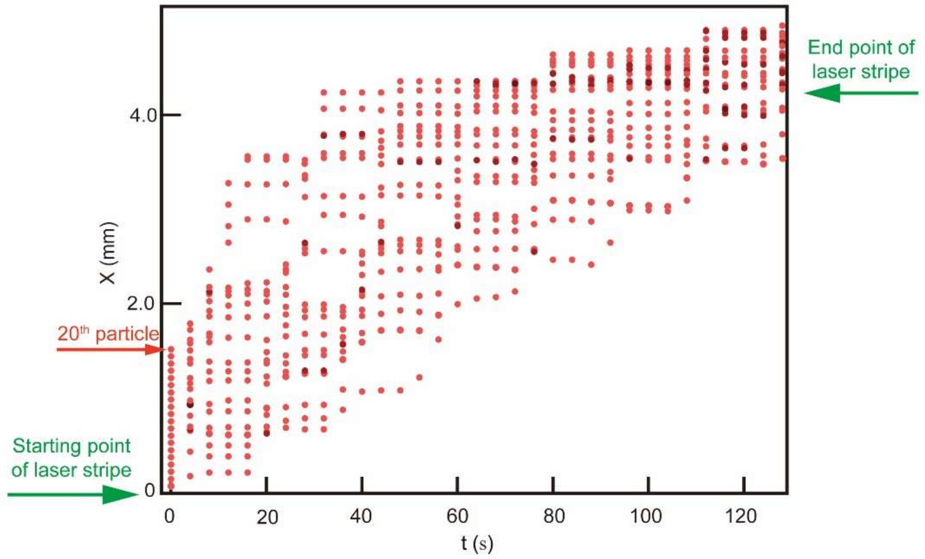

S. Fig. 13| Visualization of dynamic flow. The spatial position of particles changes as the laser strip moves. Red dots represent particles, and the shade of color represents the number of particles overlaps at that position.

Note 7. The design process of letterpress patterning.

Firstly, we convert the target pattern into a 1400×1050 data matrix, which matches the DMD's pixel size. The lines of the pattern correspond to 1, while the blank areas correspond to 0, shown in S. Fig. 14a. We then use the expansion algorithm to gradually widen the pattern's lines until all the elements in the matrix become 1. The resulting sequence of matrices is retained and labeled as $A_1, A_2, A_3 \cdots A_n \cdots A_f$, where A_1 represents the original matrix, A_n represents the matrix after the $n-1th$ expansion and A_f represents the final matrix. The corresponding pattern to A_f shown in shown in S. Fig. 14b. To obtain uniform closed stripes, we add and subtract the matrices at specific intervals, m, in a sequence, resulting in a matrix $B_1 = A_{m+1} - A_1 + A_{3m+1} - A_{2m+1} + A_{5m+1} - A_{4m+1} + \cdots$, where the matrix B_1 corresponding to a rasterized pattern shown in S. Fig. 14d. There are a total of m B-series matrices, $B_n = A_{m+n} - A_n + A_{3m+n} - A_{2m+n} + A_{5m+n} - A_{4m+n} + \cdots$. As the serial number n increases, the outer part of the stripe expands outward, while the inner part contracts inward. We fill all the holes of the target pattern, corresponding to the matrix named A_{in}, which shown in S. Fig. 14c. The pattern corresponding to matrix $C_n = (B_n -$

$A_{in}) > 0$ is the outer part of the pattern corresponding to B_n; The pattern corresponding to matrix $D_n = B_n - C_n$ is the inner part of the pattern corresponding to B_n. Each matrix corresponds to a pattern, and these patterns are like the frames in a movie, and the switching of frames brings about the change of patterns. As the serial number increases from 1 to m, the pattern lines corresponding to the C-series matrices flow outward like waves, as shown in three frames in S. Fig. 14d. Conversely, as the serial number decreases from m to 1, the pattern lines corresponding to the D-series matrices expand like flowers, as shown in three frames in S. Fig. 14e.

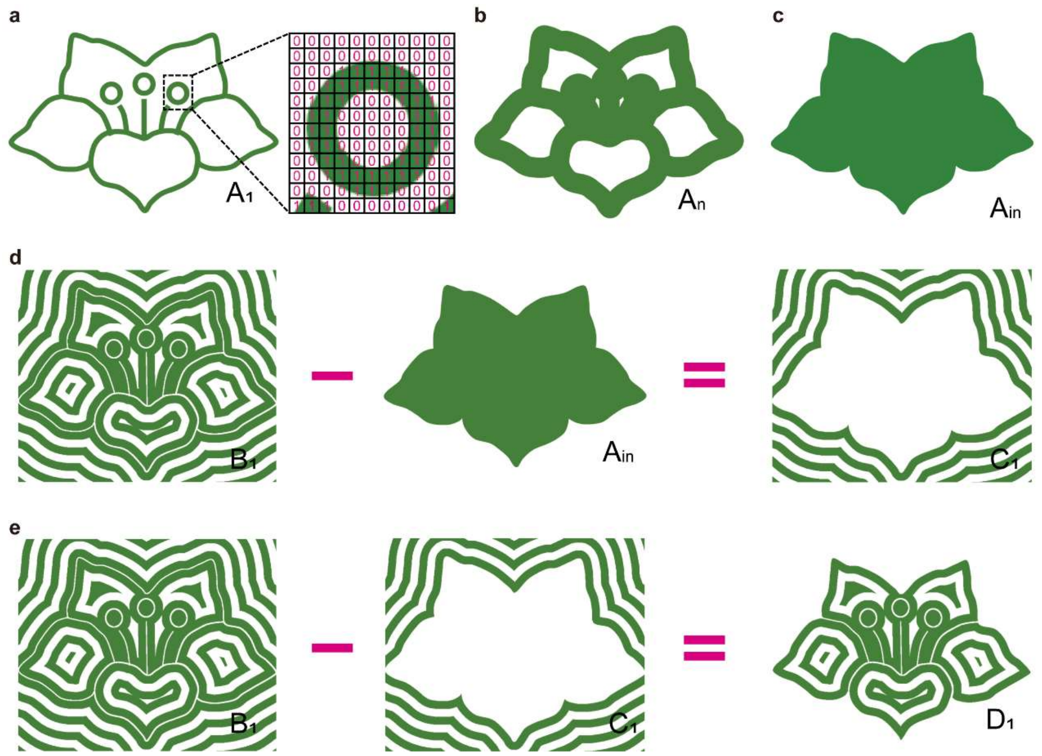

S. Fig. 14| The procedure of letterpress patterning. a, The corresponding target pattern to matrix A_1. **b**, The corresponding pattern to matrix A_n. **c**, The corresponding target pattern to matrix A_{in}. **d**, Diagram of obtaining C_1 by subtracting A_{in} from B_1. **e**, Diagram of obtaining D_1 by subtracting C_1 from B_1.

Note 8. The realization of intaglio pattern with pine flower powder

The particles employed in the main text are primarily reflective powder, which are smooth, rigid, and easily observable, providing advantages for demonstrating

experimental results. However, our method has a high degree of freedom in material selection for the manipulated particles. Pine pollen is significantly different from reflective powder in all aspects. As a natural particle, pine pollen is active and has a complex structure. The adhesion force between pine pollen and the surface of the multilayer membrane is significantly greater than that between reflective powder and the multilayer membrane. With the same laser power and target pattern (shown in S. Fig. 15a), the patterned result of pine pollen is slightly inferior to that of reflective powder, as depicted in S. Fig. 15c.

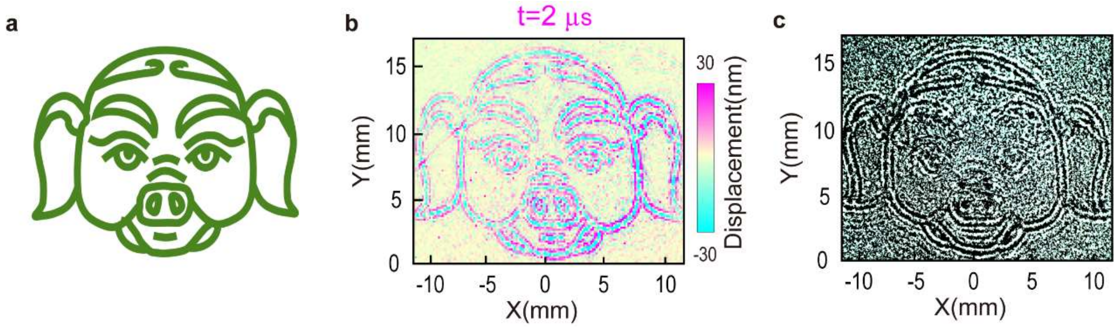

S. Fig. 15| The Pigsy pattern of pine flower powder by POAP. a, The target Pigsy pattern and the laser illuminated region, the width of green line is 400 μm. **b**, The multilayer membrane displacement of elastic wave fields at 2 μs. **c**, The pine flower powder patterning results.

Note 9. The manipulation of a few numbers of microparticles by concentric circles type light spot

The use of concentric circles as excitation sources is a common technique in various manipulation methods, as it allows vibration to be focused at the center of the circle. In our study, we apply this technique to a multilayer membrane that is thin enough to consider the excitation source and the focus point in the same plane. To create the excitation source, we use three concentric circles consisting of a circle and two rings with a period of 240 μm and a duty cycle of 50 percent. This configuration is shown in S. Fig. 16a. We consider the attenuation of pulse-excited lamb wave propagation across

the membrane and therefore opt to use only three concentric circles. We simulated the sound field and observed a circular depression in the center, as shown in S. Fig. 16b. This depression corresponds to the experimental result, which is also shown in S. Fig. 16c.

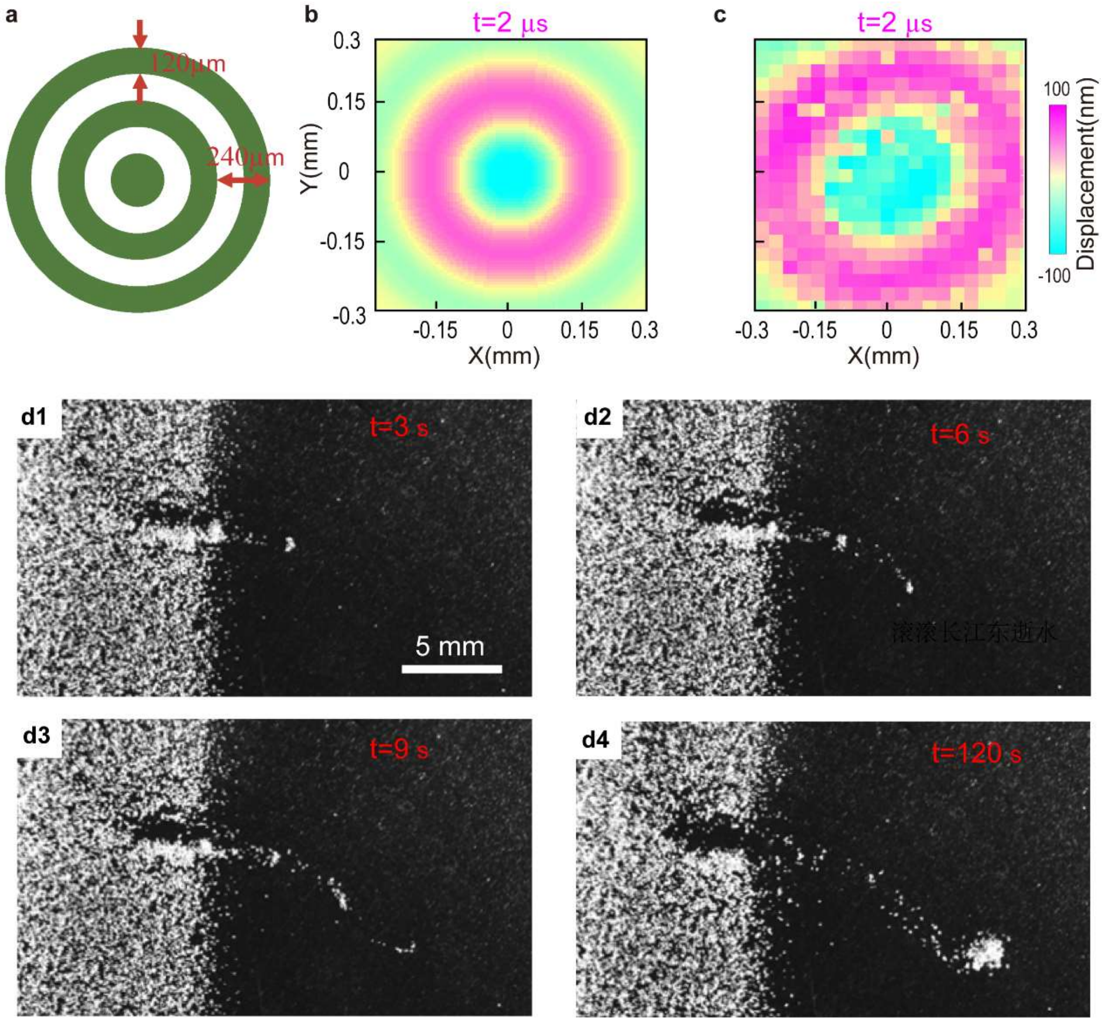

S. Fig. 16| POAP of a few numbers of microparticles. a, The diagram of concentric circles. **b**, The simulated displacement at 2 μs. **c**, The experiment displacement at 2 μs. **d**, The transportation ability of concentric circles.

Using the DMD, we can easily change the position of the concentric circles. As a result, the aggregated particles form a line that follows the same trajectory as the movement of the circles. This feature of the system allows us to precisely manipulate the position of the particles and control their behavior with high accuracy. By adjusting

the position of the concentric circles, we can create intricate shapes and patterns that have numerous applications in biotechnology, materials science, and microfabrication. The vibrations generated by the concentric circles remain confined to their own dimensions, ensuring that nearby circles do not interfere with each other.

S. Fig. 16d depicts the movement of 30 particles along a particular route, covering a distance of 15.0 mm and arriving at the target position at t=9.0 s, which indicates that the control precision of POAP is less than 100.0 μm. After 120 s, a considerable number of particles are pulled from the starting point to the endpoint, forming clusters.

Note 10. The potential application of PPAP.

The schematic of layer-by-layer transferring is shown in S. Fig. 17a. We first patterned the quartz sands with size of 150 μm on the multilayer membrane. Secondly, we brought a base plate, coated with a layer of polymer adhesive, into contact with the front side of the intaglio-patterned sands. Finally, the patterned sands on the multilayer were then transferred onto the base plate. By repeating the process, the particles were stacked layer by layer, forming a 3D printed object.

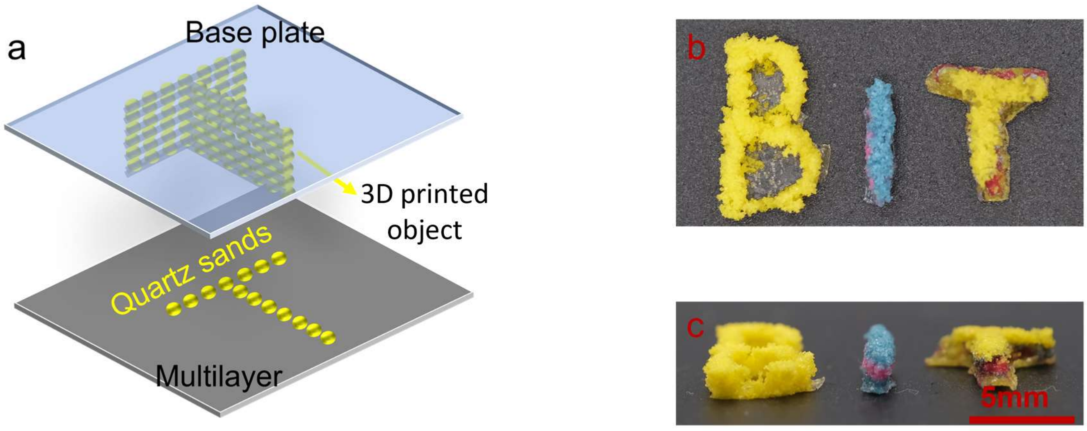

S. Fig. 17| Layer-by-layer 3D printing. a, Schematic of the photoacoustic 3D printing process. **b,** Top view of the 3D printed object. **c,** Front view of the 3D printed object.

The printing results are shown in S. Fig. 17b and c. For the structure of "I," consists of three layers. On the other hand, the structure of "T" consists of five layers. Our

method offers the flexibility to choose the material, shape, and size of the particles for each layer.

While the initial results presented in S. Fig. 17 may exhibit some discrepancies compared to commercial 3D printing systems, we have confidence that this method can be further refined to achieve a level suitable for practical applications in the future.

Supplementary References

1 Chen X., Chen Y. T., Yan M., and Qiu M. Nanosecond Photothermal Effects in Plasmonic Nanostructures, *ACS Nano* **6** (3), 2550-2557 (2012).

2 Auld, B. A., and R. E. Green. Acoustic fields and waves in solids. *Physics Today* **27**(10), 63-64 (1974).

3 Rossignol C., Rampnoux J. M., Perton M., Audoin B., and Dilhaire S. Generation and Detection of Shear Acoustic Waves in Metal Submicrometric Films with Ultrashort Laser Pulses. *Phys. Rev. Lett*. **94**, 166106 (2005).

4 Nagy P. B. and Nayfeh A. H. Viscosity-induced attenuation of longitudinal guided waves in fluid-loaded rods, *The Journal of the Acoustical Society of America*, **100**(3), 1501-1508 (1996).

5 Bowling, R.A. A Theoretical Review of Particle Adhesion. *In: Mittal, K.L. (eds) Particles on Surfaces 1. Springer, Boston, MA.* (1988).

6 Visser, J. Particle adhesion and removal: A review, *Particulate Science and Technology,* **13**(3-4), 169-196 (1995).
