# Supplementary material for: Programmable photoacoustic patterning of microparticles in air
Source: Nat Commun. 2024 Apr 16;15:3250. doi: 10.1038/s41467-024-47631-8 (PMC11021490; doi:10.1038/s41467-024-47631-8)
Supplement: Supplementary file 3 — Description of Additional Supplementary Files [file 41467_2024_47631_MOESM3_ESM.pdf]

Description of Additional Supplementary Files

File Name: Supplementary Movie 1
Description: PPAP system arranges particles into complex and precise patterns over a large area.

File Name: Supplementary Movie 2
Description: PPAP system produces letterpress style kapok flower patterning.

File Name: Supplementary Movie 3
Description: PPAP system moves about 30 silica particles along a specified path.
